# The Effect of Mesenchymal Stem Cells and Chitosan Gel on Full Thickness Skin Wound Healing in Albino Rats: Histological, Immunohistochemical and Fluorescent Study

**DOI:** 10.1371/journal.pone.0137544

**Published:** 2015-09-24

**Authors:** Abir O. El Sadik, Tarek A. El Ghamrawy, Tarek I. Abd El-Galil

**Affiliations:** Anatomy and Embryology Department, Faculty of Medicine, Cairo University, Cairo, Egypt; Cedars-Sinai Medical Center; UCLA School of Medicine, UNITED STATES

## Abstract

**Background:**

Wound healing involves the integration of complex biological processes. Several studies examined numerous approaches to enhance wound healing and to minimize its related morbidity. Both chitosan and mesenchymal stem cells (MSCs) were used in treating skin wounds. The aim of the current work was to compare MSCs versus chitosan in wound healing, evaluate the most efficient route of administration of MSCs, either intradermal or systemic injection, and elicit the mechanisms inducing epidermal and dermal cell regeneration using histological, immunohistochemical and fluorescent techniques.

**Material and Methods:**

Forty adult male *Sprague Dawley* albino rats were divided into four equal groups (ten rats in each group): control group (Group **I)**; full thickness surgical skin wound model, Group **II**: Wound and chitosan gel. Group **III**: Wound treated with systemic injection of MSCs and Group **IV**: Wound treated with intradermal injection of MSCs. The healing ulcer was examined on day 3, 5, 10 and 15 for gross morphological evaluation and on day 10 and 15 for histological, immunohistochemical and fluorescent studies.

**Results:**

Chitosan was proved to promote wound healing more than the control group but none of their wound reached complete closure. Better and faster healing of wounds in MSCs treated groups were manifested more than the control or chitosan treated groups. It was found that the intradermal route of administration of stem cells enhanced the rate of healing of skin wounds better than the systemic administration to the extent that, by the end of the fifteenth day of the experiment, the wounds were completely healed in all rats of this group. Histologically, the wound areas of group IV were hardly demarcated from the adjacent normal skin and showed complete regeneration of the epidermis, dermis, hypodermis and underlying muscle fibers. Collagen fibers were arranged in many directions, with significant increase in their area percent, surrounding fully regenerated hair follicles and sebaceous glands in the dermis of the healed areas more than in other groups.

**Conclusion:**

MSCs enhanced the healing process of wound closure more than chitosan gel treatment. Furthermore, MSCs injected intradermally, were more efficient in accelerating wound healing than any other mode of treatment.

## Introduction

Wound healing involves the integration of complex biological processes of interaction among several types of cells, intercellular matrix and signaling factors [1]. Appropriate organization of numerous aspects, such as blood clotting, inflammatory cell infiltration, cellular proliferation, neoangiogenesis and remodeling of extracellular matrix, enhances epidermal cell proliferation over dermal granulation tissue [2, 3, 4].

Numerous researches investigated the use of several strategies to promote wound healing and to minimize tissue exposure to infection and fluid losses in ulcers or burn crisis. One of these strategies was the use of growth factors such as platelet derived growth factor-BB (PDGF-BB), epidermal growth factor (EGF) and keratinocyte growth factor (KGF) [5, 6, 7, 8]. Moreover, topical application of various substances like chitosan resulted in promising effects on treatment of ulcers and wounds due to its anti-inflammatory and antibacterial properties [9]. Chitosan, a poly D-glucosamine, is a deacetylated derivative of chitin that promotes an immunomodulatory influence by enhancing cellular immune response [10]. Several studies have examined the effects of chitin and chitosan on open wound healing in multiple animal models. Chitosan was found to enhance the formation of granulation tissue, migration of inflammatory cells and early re-epithelialization. Chitosan was proved to stimulate correct assembly of collagen fibres in the extracellular matrix in wound area [11, 12]. Although, chitosan was demonstrated to accelerate the healing process, most of the results were inconclusive [13].

Engineered skin elements have proved to significantly induce advances in wound care. Nevertheless, this approach has important limitations such as their high cost, limited effectiveness, and their inability to reform skin appendages. Broad application of stem cells have provided a potential solution for the regeneration of skin wounds [14, 15, 16]. Stem cells were attributed to have several advantages as they are easily isolated from variable tissues, expand rapidly in cell culture, proliferate in a great capacity and have the ability to differentiate into different tissue types [17]. These advantages can create skin components that could not be found in the tissue engineered skin substitutes. Stem cells have distinguishing characteristics such as life span self- renewal and a great appeal for tissue regeneration in vitro and in vivo [15]. Stem cells also offer a prominent supply of functional cell lineages from pathogen-free sources for replacement of cell losses through the differentiation into adipocytes, chondrocytes, osteocytes and myoblasts [18]. Clinical studies considered mesenchymal stem cells (MSCs) an efficient source of cells in the treatment of ischemic wounds [14, 16, 19, 20].

Bone marrow derived mesenchymal stem cells (BM-MSCs), which are considered multipotent progenitor cells, are self-renewing stem cells undergoing symmetrical and asymmetrical cell divisions. Several studies have shown their potential therapeutic value in improving repair of the injured heart [21], brain [22] and cutaneous wounds in animals [15]. BM-MSCs have the capacity to promote wound healing through the release of numerous trophic factors that modulate inflammation and remodelling [23]. Stem cells enhanced the expression of vascular endothelial growth factor (VEGF) in the healing wound with acceleration of the process of angiogenesis and neovascularization of dermis [20, 24]. One of the beneficial effects of MSCs is their immunoregulatory properties through the production of multiple mediators. TSG6 was found to be an anti-inflammatory mediator produced by MSCs [25]. Moreover, TSG6, released from MSCs in acute wound healing, has anti-fibrogenic properties that enhance tissue repair [26]. BM-MSCs expressed other immunomodulatory molecules, such as TGFβ1, IDO and PGE2 when they were subjected to proinflammatory cytokines such as IL-1β, TNFα and INFα-2b [27]. The immunomodulatory factors; COX2, PTGES and TSG6, upregulated by IL1beta, were found to reduce post injury inflammation and accelerate wound healing [28].

Adipose derived mesenchymal stem cells (ASCs) can promote wound healing through macrophage stimulating protein release [29]. **Hattori and Ishihara** [30] found that ASCs secreted higher quantities of angiogenic growth factors such as VEGF, HGF and IGF-1when injected intradermally into an irradiated mouse, than bone marrow derived stromal cells. ASCs—conditioned medium was proved to be a novel therapy for hair regeneration [31]. Self-assembled ASC spheroids grown on chitosan-hyaluronan membranes, applied in a designed rat skin repair model, expressed more cytokine genes (VEGF and FGF1) and migration associated genes (MMP1 and CXCR4) compared with ASCs dispersed single cells. The expression of cytokine genes might attribute enhanced migration of keratinocytes with increased homing of ASCs. These effects resulted in reepithelialization and angiogenesis. ASCs spheroids promoted wound closure with a significant higher ratio of angiogenesis through paracrine effects [32]. Transplantation of low-level light irradiated ASC spheroids in the wound bed revealed that ASC spheroid expressed angiogenic factors that increased the density of vascular formation and differentiated into endothelial cells [33].

The present study aims to compare MSCs effects versus chitosan in wound healing, evaluate the route of administration of mesenchymal stem cells and highlight the possible mechanisms that induce cell regeneration for promotion of wound healing.

## Material and Methods

### Preparation of chitosan gel

In order to prepare 1% Chitosan solution, 1 g of low molecular weight Chitosan (Sigma, USA) was dissolved in 100 ml of 1% (v/v) aqueous acetic acid and mixed for 4 hours [34].

### Isolation, culture and labeling of MSCs

MSCs were obtained from the Biochemistry and Molecular Biology Unit, Faculty of Medicine, Cairo University. Bone marrow stromal cells were obtained from femurs and tibiae of albino rats by aspiration. They were isolated by flushing the bone marrow cavity with Dulbecco’s Modified Eagle’s Medium (DMEM) (Sigma, USA, D5796) supplemented with 10% fetal bovine serum (FBS) (Sigma, USA, F6178). The cells were layered over Ficoll-Hypaque (Sigma, USA, F8016) in a ratio of 2:1 in sterile conical tubes and centrifuged. The opaque layer containing the mononuclear cells was aspirated and suspended in culture medium supplemented with 1% penicillin-streptomycin (Sigma, USA, P4333) and incubated at 37°C in 5% humidified CO_2_ for 14 days and the media were changed every 3–4 days. When large colonies developed (80–90% confluence), cultures were washed twice with phosphate buffer saline (PBS) (Sigma, USA, P5493) and cells were trypsinzed with 0.25% trypsin (Sigma, USA, T1426) in 1ml Ethylene Diamine Tetra Acetate (EDTA) (Sigma, USA, E6758) for 5 minutes at 37°C. After centrifugation (at 2400 rpm for 20 minutes), cell pellets were resuspended with serum supplemented medium and incubated in 25cm^2^ culture flasks. The resulting cultures were referred to first passage cultures [35].

### Immunophenotyping of the separated cells

MSCs were washed and resuspended in phosphate-buffered saline. CD29 (Sigma, USA, SAB4501582) and CD45 (Sigma, USA, OX-1 84112004), monoclonal antibodies were added directly to cells and kept for 1 hour in 4°C. The cells were then incubated with antimouse immunoglobulin G fluorescein conjugated secondary antibody (Millipore Corp, Temecula, CA) for 45 minutes on ice. Cell suspensions were washed twice and analyzed on a FACS caliber flow cytometer [36, 37].

### Labeling of MSCs with PKH26 dye and cell viability analysis

Culture cells were labelled with fluorescent cell tracker PKH26 (Sigma, USA, MINI26) according to manufacturer’s instructions [38]. Cell viability was detected by adding 1:1 ratio of cell suspension and 0.4% trypan blue stain, and examined under the phase contrast microscope. Viable cells appeared shiny without staining [39].

### Animals

Forty adult male *Sprague Dawley* albino rats with an average weight of 200–250 g were used in this study. They were locally bred at the animal house at Faculty of Medicine, Cairo University, Egypt. The animals were housed at room temperature and had access to food and water ad libitum. The animals were given two weeks acclimatization period before starting the experiment. They were treated in accordance with the international guidelines for the care and use of laboratory animals and the experiment protocol was approved by the Ethics Committee, Faculty of Medicine, Cairo University. This included the way of animal treatment, anaesthesia and induction of full thickness surgical skin wound, methodology of collecting the MSCs from the bone marrow of animals and their use in experimental research. All the efforts to ensure minimal animal sufferings were taken.

### Induction of full thickness cutaneous wound healing model and evaluation of its healing process

Each rat was anesthetized by an intramuscular injection of ketamine and xylazine, at dose of 40 and 5mg/kg body weight, respectively [13]. The surgical area was shaved with an electric razor and disinfected using 70% ethanol. After a deep surgical plane of general anesthesia had been reached, a square wound (1cm^2^) was performed on the middle of the right dorsal side of all groups. Both the epidermal and dermal layers were removed down to the subcutaneous connective tissue, creating a full-thickness wound. The wounds were then covered with a Curity® non-adhering dressing, bandage with a Curiy® Sheer Bandage and allowed to heal. The bandages were replaced every other day.

The animals were divided into 4 equal groups of 10 rats each:

- **Group I** (control group). Ten wounded rats received 0.5 ml phosphate buffered saline (PBS) (P5493, Sigma, USA) injections only without MSCs. Half of them received it intradermally and the other half received it by systemic injections.
- **Group II** (chitosan gel treated group). 0.5 ml of chitosan gel was applied for ten wounded rats once daily, till the end of the experiment, after cleaning the wound with a dilute solution of Dettol®.
- **Group III** (MSCs systemically treated group). Ten wounded rats, each of them received one systemic injection (through the caudal vein) of MSCs (1 x 10^6^) diluted in 0.5 ml of PBS at the day of induction of the full thickness surgical skin wound [4].
- **Group IV** (intradermal MSCs treated group). Ten wounded rats, each of them received MSCs (1 x 10^6^) diluted in 0.5 ml of PBS. They were injected intradermally, using BD Ultra-Fine™ II Short Needle Insulin Syringe, Gauge 31, BD, USA, at 4 injection sites in the margin of the surgical skin wound, each side received 0.25x10^6^ cells. A silicone circular patch 2.5 cm in diameter was placed on an area covering the wound and enough healthy skin around, then the patch was fixed at the margin to the healthy skin with skin adhesive (Leukosan ® Adhesive, BSN medical). The wounds were then covered with a Curity® non-adhering dressing, bandage with a Curiy® Sheer Bandage and allowed to heal.

The animals were housed individually to avoid infection or further damage to the wounds by the others.

### Gross evaluation of wound healing

Wound contraction was calculated at the 3^rd^, 5^th^, 10^th^ and 15^th^ days after wound induction. The percentage of wound healing was calculated as performed by [8, 40]:

area of original wound − area of actual wound/area of original wound × 100

Progressive decrease in wound area was measured periodically by tracing the wound margin on a tracing paper. The tracing was then placed onto 1 mm² graph sheet paper and the numbers of squares were counted. The total open wound area at each tracing was subtracted from that of the initial tracing to determine the area of contraction and repithelization during the period since wounding [41].

### Microscopic evaluation of wound healing

The animals were re-anaesthetized using 75mg/kg sodium thiopental, then full thickness skin specimens from the entire wound with safety margin of 0.5 cm of healthy skin around the healing wound were excised at 10^th^ and 15^th^ days after wound induction from each group. Then, they were rapidly fixed in 10% formal saline solution, processed, embedded to obtain paraffin blocks and cut at 5–6 micron thickness sections. Sections were stained with Haematoxylin and Eosin and Masson’s trichrome for studying the collagen fibers in the healing granulation tissue [42].

### Immunohistochemical staining with CD31

The sections were collected on poly-L-lysine coated slides and nonspecific endogenous peroxidase activity was blocked by treatment with 0.9% hydrogen peroxide in absolute methanol for 10 min. Then antigen retrieval was performed by heating the sections in 10 mM sodium citrate buffer, in a water bath at 95–100°C for 30 minutes. Sections were rinsed twice in PBS Tween 20 for 2 minutes, then blocked with 5% normal goat Serum for 30 minutes at room temperature. They were incubated with the primary antibody for 30 minutes, CD31 mouse monoclonal IgG1 antibody, Catalog # MA5-13188; dilution 1:100) as an endothelial cell marker for detecting the neoangiogenesis process. A biotinylated goat anti-polyvalent secondary antibody, Catalog **#** TP-060-BN was applied for 60 min at room temperature. Immunodetection was carried out with the horseradish peroxidase-avidin-biotin complex method using a VECTASTAIN® Elite ABC kit (Vector Laboratories Inc., Burlingame, CA) and DAB was applied as the chromogen. Localization was visualized with DAB and counter-stained in Meyer’s hematoxylin, dehydrated, and mounted. Negative control sections were performed with the same procedure mentioned before except that the primary antibody was replaced by non-immune mouse serum. All antibodies were purchased from Thermo Fisher Scientific, USA [43].

**Fluorescence detection** (PKH26-labeled MSCs) by fluorescent microscope in unstained paraffin sections.

### Morphometric measurements and statistical analysis

The following parameters were measured:

1. Epidermal thickness was measured in H & E stained sections.
2. The area percent of collagen fibers in Masson’s trichrome stained sections.
3. The area percent of immune reaction for CD 31.

The measurements were taken by an independent observer, who was unaware of the experimental design. They were obtained in ten non overlapping fields per specimen at a magnification of 400 by using Leica LAS V3.8 image analyzer computer system (Switzerland). The data obtained for all groups were expressed as mean and standard deviation (±SD) and subjected to statistical analysis using "SPSS 22" software. One-way analysis of variance (ANOVA) for comparison between the different groups at each time point, day 10 and 15, was done. In order to compare the same group values between day 10 and 15, paired sample "T" test was performed. Results were considered significant when p value was ≤0.05 [44].

## Results

### Gross evaluation of wound healing

In all groups, the induced wounds expanded after surgery to variable extents. By the 3^rd^ day all wounds had an area that exceeded 1.5 cm^2^ (S1 Table and S1 Chart).

#### Group I (control group)

The wound area expanded and reached a mean value 2.34 cm^2^ on the 3^rd^ day after surgery. The wound area decreased slowly from the fifth day onwards (0.9% reduction after 10 days and 30% reduction after 15 days). However complete closure could not be detected in any rat till the end of experiment.

#### Group II (chitosan gel treated group)

The wound area expanded and reached a mean value of 2.12 cm^2^ on the 3^rd^ day after surgery. The wound area decreased from the fifth day onwards in all rats with a relatively rapid rate compared with the control group (13.1% reduction after 10 days and 50% reduction after 15 days). However, complete closure could not be detected also in any rat till the end of experiment.

#### Group III (MSCs systemically treated group)

The wound area expanded and reached a mean value 1.8 cm^2^ on the 3^rd^ day after surgery. The wound area decreased from the fifth day onwards in all rats with a relatively rapid rate compared with the control group and the chitosan gel treated group (28.2% reduction after 10 days and 96.1% reduction after 15 days). By the end of the fifteenth day, the wounds were completely closed in 8 out of 10 rats.

#### Group IV (intradermal MSCs treated group)

The wound area expanded and reached a mean value 1.7 cm^2^ on the 3^rd^ day after surgery. This group showed the most rapid rate of wound healing (40.3% reduction after 10 days and 100% reduction after 15 days). Thus, by the end of the fifteenth day, the wounds were completely closed in all rats.

### Histological results

#### (A) Haematoxylin and Eosin stain

**Fig 1:**
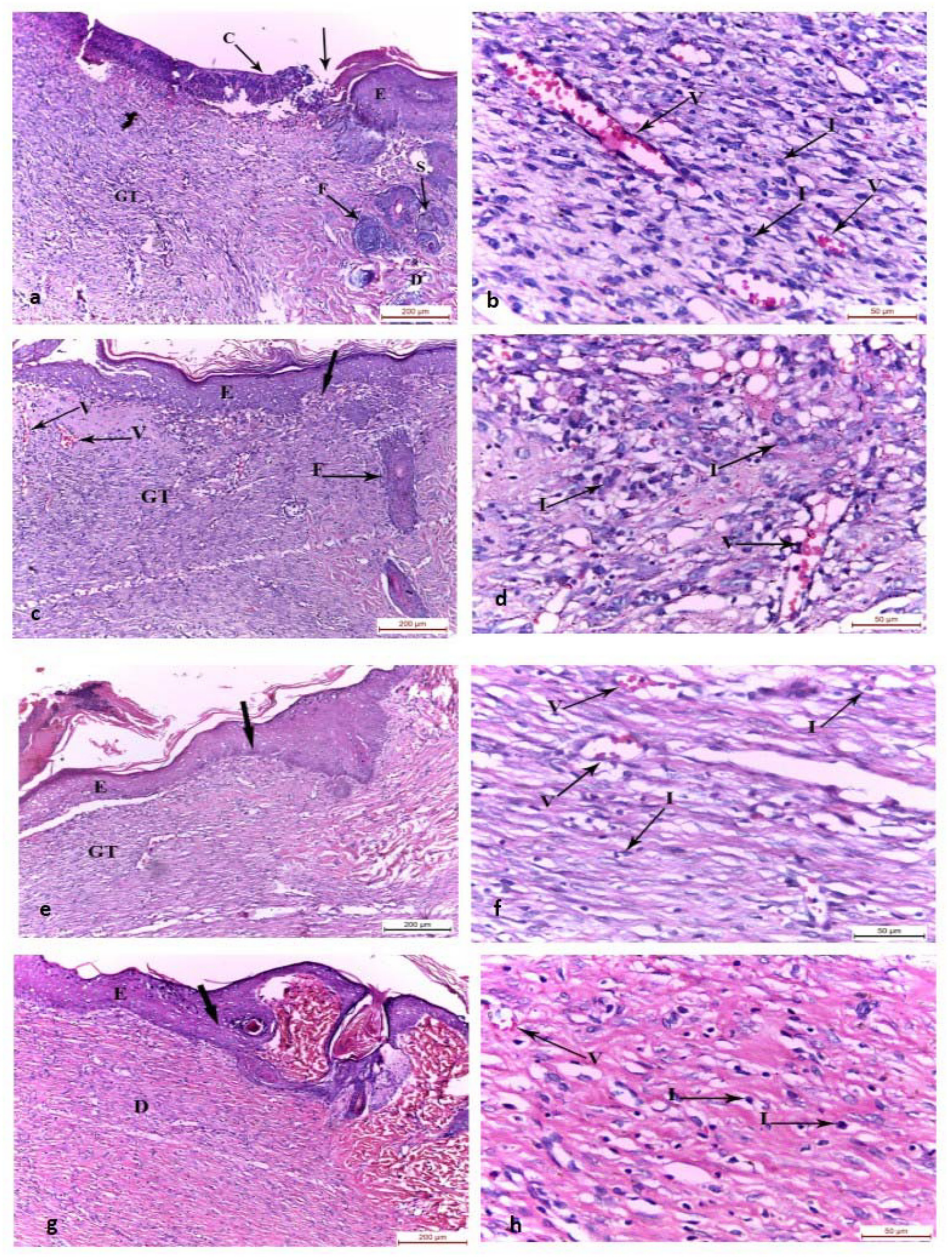
Photomicrographs of the skin wound area on the 10th day. a,b) control group showing wound surface covered by crust and the wound bed was filled with granulation tissue with absence of the epidermis and skin appendages. The adjacent normal skin showed normal epidermis, dermis, hair follicles and sebaceous gland. Wound granulation tissue showed inflammatory cell infiltrate and congested blood vessels. c,d) Group II showing wound surface covered with regenerated epidermis and wound bed filled with granulation tissue with inflammatory cell infiltrate, congested blood vessels and absence of skin appendages. e,f) Group III and g,h) Group IV showing wound surface covered by regenerated epidermis with increasing thickness than Group II and the wound bed was filled with granulation tissue with absence of skin appendages. Mild inflammatory cell infiltrate and few blood vessels were found in the dermis of wound bed compared to Group I and II. C: crust, GT: granulation tissue, E: epidermis, D: dermis, F: hair follicles, S: sebaceous gland, I: inflammatory cell infiltrate, thick arrow: junction between the normal skin and the wound area, V: blood vessel, (H&E, a-c-e-g x100; b-d-f-h x400)

The wound surface in the control group was covered by crust on the tenth day of the experiment. The wound bed was filled with granulation tissue with absence of epidermis and skin appendages. The adjacent normal skin showed normal epidermis, dermis, hair follicles and sebaceous gland (Fig 1a). The granulation tissue in the wound bed was filled with inflammatory cell infiltrate and congested blood vessels (Fig 1b). Group II (chitosan gel treated group) wound area was covered by regenerated epidermis and the wound bed was filled with granulation tissue and congested blood vessels with absence of skin appendages. The adjacent normal skin showed hair follicles (Fig 1c). Similar to the control group, group II had granulation tissue in the wound bed filled with inflammatory cell infiltrate and congested blood vessels (Fig 1d). In group III (MSCs systemically treated group), the wound area was covered by regenerated epidermis and the wound bed was filled with granulation tissue with absence of skin appendages (Fig 1e). There was mild inflammatory cell infiltrate in the dermis of the wound bed and few blood vessels (Fig 1f). In group IV (intradermally injected MSCs), the wound surface was covered by regenerated epidermis and its wound bed was filled with regenerated dermis with absence of skin appendages (Fig 1g) with mild inflammatory cell infiltrate in the dermis of the wound bed and few blood vessels (Fig 1h).

**Fig 2:**
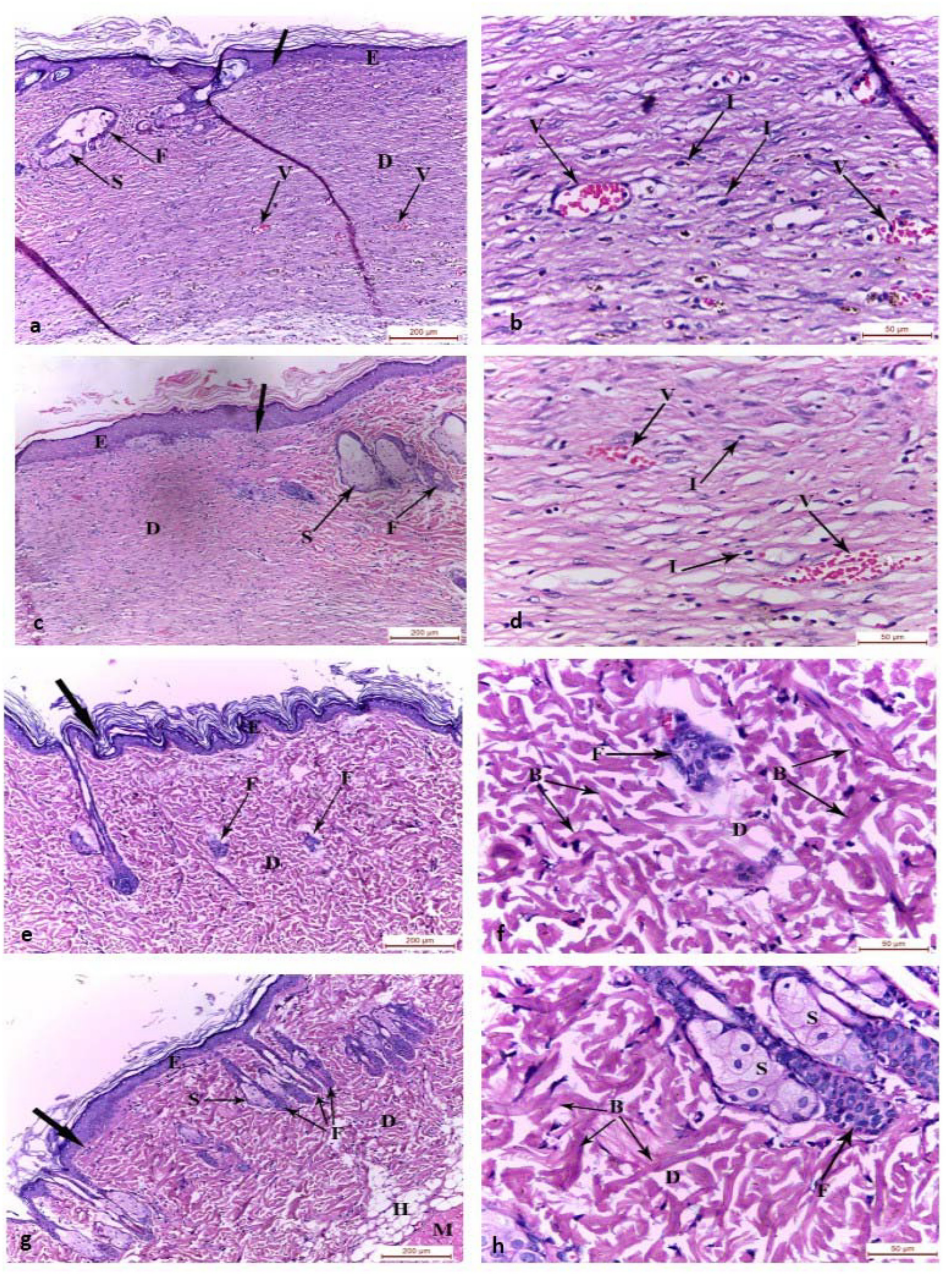
Photomicrographs of the skin wound area on the 15th day. a,b) control group showing the wound area covered by regenerated epidermis. The regenerated dermis was filled with inflammatory cell infiltrates and many congested blood vessels with absence of hair follicles and sebaceous glands. The adjacent normal skin showed hair follicles and sebaceous gland. c,d) Group II showing the wound covered by regenerated epidermis on the granulation tissue in the regenerated dermis with absence of hair follicles and sebaceous glands. The adjacent normal skin showed hair follicles and sebaceous gland. Mild inflammatory cell infiltrates appeared in the dermis of the wound bed and congested blood vessels. e,f) Group III and g,h) Group IV wound area showing complete regeneration of the epidermis and dermis. The regenerated dermis showed fully regenerated hair follicles and sebaceous glands with absence of inflammatory cells and congested vessels. Connective tissue bundles were arranged in different directions. GT: granulation tissue, E: epidermis, D: dermis, H: hypodermis, M: muscle fibers, F: hair follicles, S: sebaceous gland, I: inflammatory cell infiltrate, B: collagen bundles, thick arrow: junction between the normal skin and the wound area, V: blood vessel, (H&E, a-c-e-g x100; b-d-f-h x400)

On the fifteenth day, the control group (group I) and chitosan gel treated group (group II) showed regenerated epidermis covering the wound surface. The regenerated dermis of both groups showed congested blood vessels with absence of hair follicles and sebaceous glands (Fig 2a and 2c). The dermis of the wound bed was filled with inflammatory cell infiltrate and many congested blood vessels in the control group (Fig 2b), while it expressed mild inflammatory cell infiltrate and congested blood vessels in chitosan gel treated group (group II) (Fig 2d). The wound area in MSCs systemically treated group (group III) revealed complete regeneration of the epidermis with newly regenerated hair follicles (Fig 2e). The connective tissue bundles were loosely arranged in many directions in the dermis (Fig 2f). In group IV (intradermally injected MSCs), the wound area was hardly demarcated from the adjacent normal skin. The wound area showed complete regeneration of the epidermis, dermis, hypodermis and underlying muscle fibers. The regenerated dermis showed fully regenerated hair follicles and sebaceous glands (Fig 2g). Connective tissue bundles were arranged in many directions surrounding fully regenerated hair follicles and sebaceous glands in the dermis (Fig 2h).

#### (B) Masson’s trichrome stain

**Fig 3:**
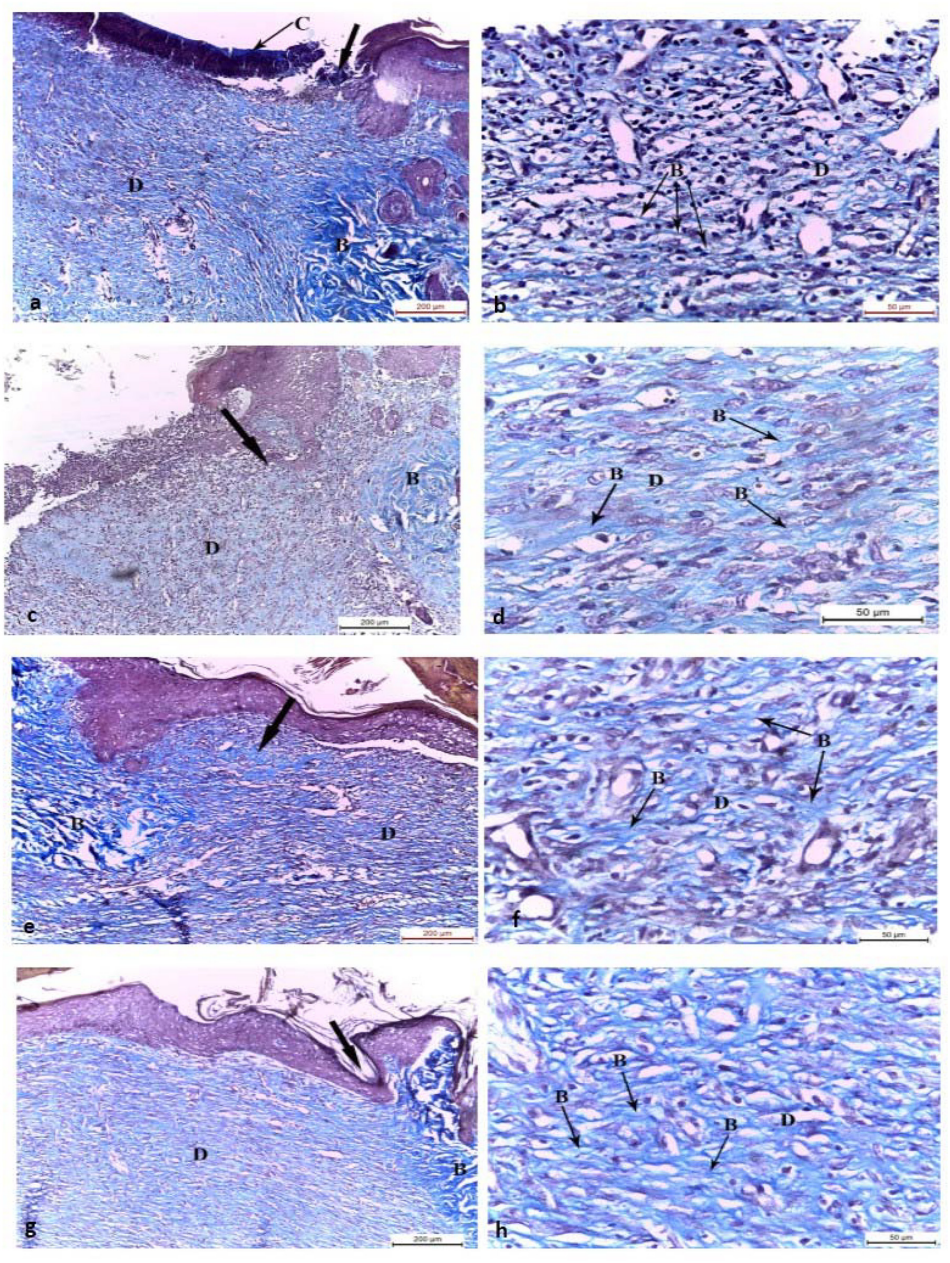
Photomicrographs of the skin wound area on the 10th day. a,b) control group showing wound surface covered by crust and the wound bed is filled with scanty collagen bundles arranged in one direction in the regenerated dermis. The adjacent normal skin showed normal collagen bundles arranged in different directions. c,d) Group II showing wound bed filled with prominent deposition of thin collagen bundles arranged in one direction, parallel to the epidermis in the regenerated dermis. e,f) Group III showing wound bed filled with prominent deposition of collagen bundles arranged parallel to the epidermis within the regenerated dermis. g,h) Group IV showing wound bed filled with prominent deposition of thick collagen bundles in the regenerated dermis arranged parallel to the epidermis. The adjacent normal skin showed normal collagen bundles arranged in different directions. C: crust, D: dermis, B: collagen bundles, thick arrow: junction between the normal skin and the wound area, (Masson's trichrome, a-c-e-g x100; b-d-f-h x400)

On the tenth day of the experiment, the wound bed of the control group (group I) and chitosan gel treated group (group II) showed scanty collagen bundles in the regenerated dermis. The collagen bundles were arranged in one direction parallel to the epidermis. The adjacent normal skin showed normal collagen bundles arranged in different directions (Fig 3a, 3b, 3c and 3d). In MSCs systemically treated group (group III) and intradermally injected MSCs group (group IV), the wound bed showed prominent deposition of collagen bundles, in the regenerated dermis, arranged parallel to the epidermis. The adjacent normal skin showed normal collagen bundles arranged in different directions (Fig 3e, 3f, 3g and 3h).

**Fig 4:**
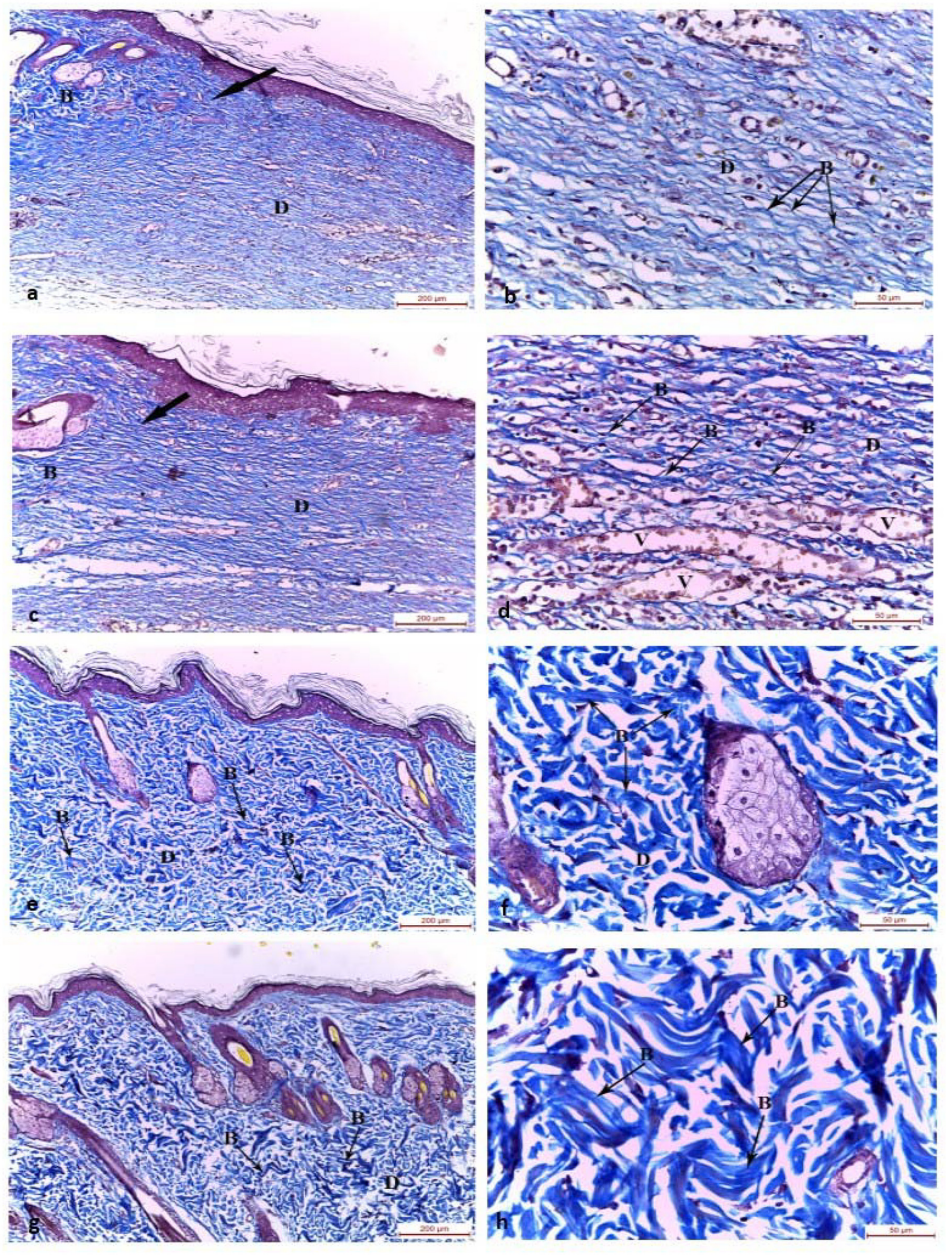
Photomicrographs of the skin wound area on the 15th day. a,b) control group showing the junction between the normal skin and the wound area. The wound bed was filled with collagen bundles arranged in one direction parallel to the epidermis in the regenerated dermis. The adjacent normal skin showed normal collagen bundles arranged in different directions around sebaceous glands and hair follicles. c,d) Group II wound bed was filled with collagen bundles arranged parallel to the epidermis in the regenerated dermis. The blood vessels are congested. The adjacent normal skin showed normal collagen bundles arranged in different directions. e,f) Group III and g,h) Group IV showing wound bed filled with prominent deposition of dense thick collagen bundles arranged in different direction in a meshwork pattern. D: dermis, B: collagen bundles, thick arrow: junction between the normal skin and the wound area, (Masson's trichrome, a-c-e-g x100; b-d-f-h x400)

On day 15, the wound bed of the control group (group I) and chitosan gel treated group (group II) were filled with collagen bundles in the regenerated dermis arranged parallel to the epidermis. The adjacent normal skin showed normal collagen bundles arranged in different directions in a meshwork pattern (Fig 4a, 4b, 4c and 4d). In MSCs systemically treated group (group III) and intradermally injected MSCs group (group IV), the wound beds were filled with dense thick collagen bundles arranged in different directions in a network like manner in the regenerated dermis (Fig 4e, 4f, 4g and 4h).

#### (C) PKH26 fluorescence stain

**Fig 5:**
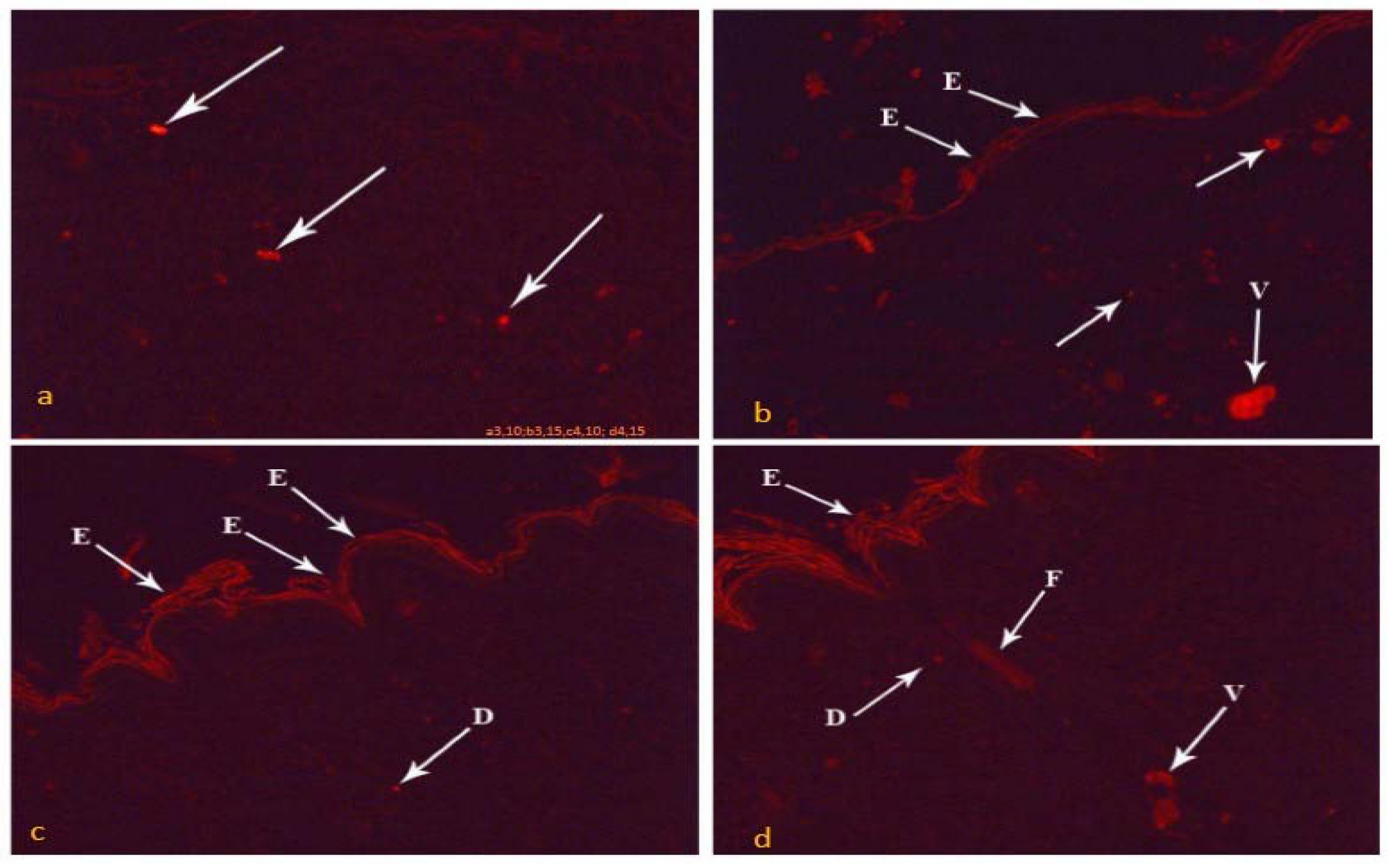
Photomicrographs of the skin wound area on the 10^th^ day. a) Group III showing red fluorescent cells (arrows) within the proliferated granulation tissue in the ulcer. b) Group IV showing fluorescent cells (arrows) within the proliferated granulation tissue, the new regenerated epidermis (E) and new blood vessels (V). By the 15th day: c) Group III showing increasing area of fluorescent cells within the regenerated epidermis (E) and within the regenerated dermis (D) than day 10 groups. d) Group IV showed the highest distribution than previous groups of fluorescent cells within the regenerated epidermis (E), the regenerated dermis (D), blood vessels (V) and hair follicle (F). (PKH, x100)

On the 10th day, MSCs systemically treated group (group III) showed PKH labelled red fluorescent cells within the proliferated granulation tissue of the wound area (Fig 5a). Intradermally injected MSCs group (group IV) had red fluorescent cells within the granulation tissue, in addition to the new regenerated epidermis and the new blood vessels within the wound area (Fig 5b). By the 15^th^ day, group III revealed increased labelled fluorescent cells compared to day 10 within the regenerated epidermis in addition to its dermis (Fig 5c). Group IV had the highest labelled cells within the regenerated epidermis, dermis, blood vessels and hair follicles, compared to the other group (Fig 5d).

#### (D) Immunohistochemistry

**Fig 6:**
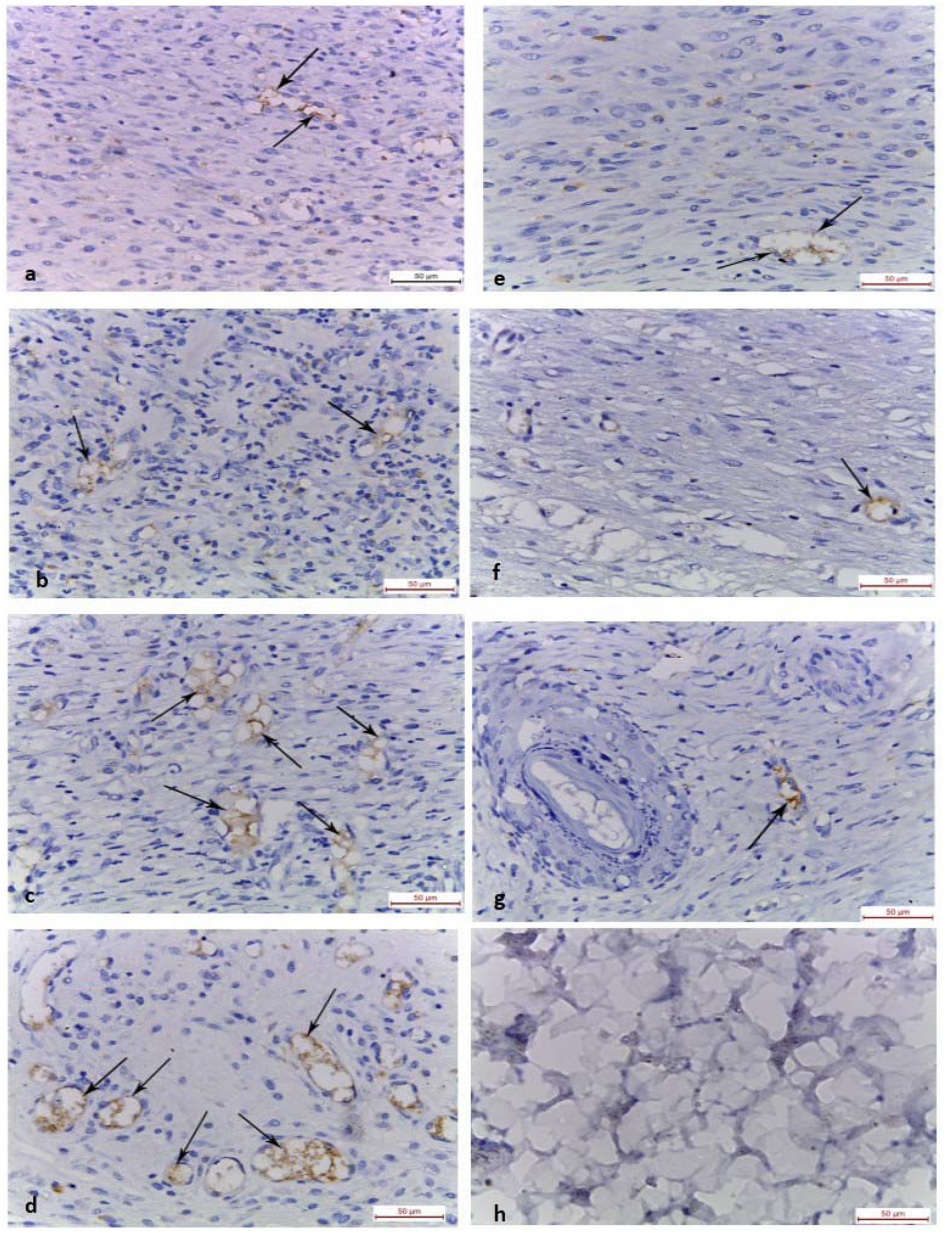
Photomicrographs of the skin wound area, day 10. (a) Control group, showing mild localized immunoreactivity in the endothelial cells of dermal blood vessels (arrows). (b) Group II showing moderate immunoreactivity. (c,d) Group III and IV showing dense immunoreactivity in the endothelial cells of dermal blood vessels (arrows). By day 15: (e,f,g) Control, Group II and III showing mild localized immunoreactivity in the endothelial cells of dermal blood vessels (arrows). (h) Group IV showing absence of immunoreactivity in the healed wound area. (CD 31; x 400)

On the 10th day, the control group (group I) showed mild localized CD31 immunoreactivity in the endothelial cells of dermal blood vessels (Fig 6a). However, chitosan gel treated group (group II) showed moderate immunoreactivity in the endothelial cells of dermal blood vessels (Fig 6b). Dense immunoreactivity in the endothelial cells of dermal blood vessels was seen in MSCs systemically treated (group III) (Fig 6c). Intradermally injected MSCs group (group IV) showed widespread dense immunoreactivity in the endothelial cells of dermal blood vessels and fibroblastic cells (Fig 6d). By the 15^th^ day, the control group, group II and III revealed mild localized immunoreactivity in the endothelial cells of dermal blood vessels (Fig 6e, 6f and 6g). Remarkably group IV showed absence of immunoreactivity in the healed wound area (Fig 6h).

#### (E) Histomorphometric results

The epidermal thickness on day 10 significantly increased in all groups compared to the control group. While, by day 15, it significantly decreased in group III and IV compared to the control group. By comparing each group on both 10^th^ and 15^th^ day, the epidermal thickness of the control group significantly increased on day 15, however for the rest of groups it significantly decreased with time (S2 Table and S2 Chart).

The area percent of collagen fibres on both the 10^th^ and 15^th^ days were significantly increased in group III (MSCs systemically treated) and group IV (intradermally injected MSCs group) compared with the control group (S3 Table and S3 Chart). The area percent of CD31 on the 10^th^ day was significantly increased in both groups III and IV in relation to the control group, however, it significantly decreased on day 15 only in group IV (S4 Table and S4 Chart). By comparing the mean values within each group on day 10 and 15, collagen fibres were significantly increased in all groups with increasing healing time. However, CD31 showed non-significant differences in both group I and II and significantly decreased on day 15 in group III and IV (S3 and S4 Tables).

## Discussion

Skin wounds from trauma, burns or chronic ulcers remained a major international health crisis particularly in developing countries. They had significant morbidity and mortality due to lack of reliable therapies in addition to other factors, including infection and tissue fluid loss. Therefore, in this study, an attempt was done to estimate promising therapies in the field of tissue repair and regeneration particularly wound healing. The rodent excision wound model was used to study skin regeneration therapies. The rate of wound closure, degree of epithelialization, collagen formation, angiogenesis and inflammatory reaction were the principal parameters that determined wound healing and contraction. Comparison was done, in the present work, among healing effects of chitosan and two delivery methods of injection of MSCs; systemic and intradermal injections.

In the present study, chitosan was proved to promote wound healing through gross evaluation of the wound size. The wound area decreased in a rapid rate compared to the control group. These results were similar to those recorded in previous studies [9, 13]. They reported that the enhancement of wound healing process of chitosan resulted from the degradation of chitosan into the active N-acetyl-d-glucosamine. Moreover, in the present work, chitosan treated wounds showed better immunoreactivity in the endothelial cells of dermal blood vessels compared with the control group. In agreement with the present study, **Alsarra** [13] concluded that these effects resulted from the catalysis of chitosan by the lysosomes transported to the wound areas by the inflammatory polymorphonuclear leucocytes. Moreover, similar results were found recently by other researchers [45, 32 **]**. They attributed that the efficiency of wound healing was affected by the wet environment maintained by chitosan gel which fastened wound repair.

MSCs have been considered to be efficient in the healing of ulcerative wounds [16, 20, 46]. They could be easily derived from bone marrow, umbilical cords or adipose tissue. They are multipotent differentiating into multiple types of cells. PKH26 was chosen to label MSCs in the current study. In accordance to several researches, PKH26 is a rapid simple labeling procedure that provides bright staining without affecting cell or protein function [47, 48]. Moreover, the local environment of skin wound affected the migration and differentiation of multipotent stem cells and that PKH26 labelled cells could be found in the regenerated hair follicles, sebaceous and sweat glands [49, 50]. They may exert paracrine effect on the formation of these structures which is consistent with **Li et al**. [51]. They found that bone marrow adherent cells containing MSCs and CD34 cells, injected intravenously, contributed to cells in hair follicles, sebaceous glands, and blood vessels in the dermis of full-thickness skin wounds.

The present work demonstrated better and faster healing of wounds in MSCs treated groups more than the control or chitosan treated groups, using both gross and microscopic evaluation. These findings could be attributed to the effects of MSCs through the detection of red fluorescent of PKH26 labeled MSCs in the regenerated wound areas. These results are consistent with those reported by **Dai et al**. [52]. They found that umbilical cord derived MSCs enhanced skin tissue regeneration in vivo and could be considered an ideal cell source for therapy of skin wounds and burns. Furthermore, earlier reduction of wound area and wound closure was reported in BM-MSCs injected groups [20, 53]. These findings might be attributed to wound contraction resulting from the differentiation of BM-MSCs into myofibroblasts and fibroblasts. In addition, BM-MSCs possess chemotactic effects recruiting endogenous fibroblasts from the surrounding tissues [54, 55]. Also in agreement with the current study, ASCs demonstrated acceleration to the healing of ulcers when cultured in vitro then allowed for allogenic transplantation. They could survive for 14 days allowing cell tracking and proliferate in the ulcers site creating long term influences in wound healing. ASCs could either fuse with the endogenous endothelial cells of the host or differentiate into exogenous endothelial cells [56, 57].

The route of delivery of stem cells influenced the rate and efficiency of wound healing. It was found, in this study, that the intradermal route of administration of stem cells enhanced the rate of healing of skin wounds better than the systemic administration to the extent that, by the end of the fifteenth day of the experiment, the wounds were completely closed in all rats. Histologically, the wound areas were hardly demarcated from the adjacent normal skin and showed complete regeneration of the epidermis, dermis, hypodermis and underlying muscle fibers. Collagen fibers were arranged in many directions indicating maturation in their arrangement, with significant increase in their area percent, surrounding fully regenerated hair follicles and sebaceous glands in the dermis of the healed areas. This enabled the dermis to afford greater forces applied upon. Moreover, intradermally injected stem cells group had the highest PKH26 labelled cells within the regenerated epidermis, dermis, blood vessels and hair follicles, compared to the systemically injected group. This signified their engraftment into the wound area and their differentiation into different skin components.

The current results showed widespread significant dense immunoreactivity of CD31 in the endothelial cells of dermal blood vessels and fibroblastic cells, on day 10, in the intradermally injected stem cells group, indicating marked endothelial cell proliferation and neoangiogenesis formation process compared to the other groups. The remarkable absence of immunoreactivity in the healed wound area of this group on day 15 clearly demonstrated the complete healing process and their higher therapeutic efficiency in cutaneous healing than other modes of treatment tested in this experiment. These findings were in accordance with several recent studies. **Wu et al**. [20] injected GFP positive BM-MSCs in the wound areas. They found significant acceleration of wound closure, early epidermal regeneration, increased cellularity and angiogenesis. **Karp and Teo** [58] explained the faster and better results of topical injection of BM-MSCs in wound healing. They recorded that the systemic injection of BM-MSCs mimicked the route of endogenous MSCs in the systemic circulation to the target sites. They found, through cell tracking, that the stem cells could be trapped in the liver, lungs and spleen. These findings could explain the delay or the reduction of the numbers of cells that finally reached the sites of skin wounds. The histological influences of human cord blood MSCs on wound healing in diabetic mouse after topical and systemic injection was examined. There was an acceleration in wound healing which was statistically significant in the topical injection models but non-significant in the systemically injected models [59]. The authors suggested that there was a direct contribution of BM-MSCs in wound regeneration through the expression of keratin which is a keratinocyte-specific protein. ASCs injected into the wound site had better and faster wound healing in the topically injected ASCs [4] which is in agreement with the results of the present study.

### Conclusion

The present work suggested that wound healing could be accelerated by several methods. Chitosan gel was found to enhance wound healing. However, MSCs showed even higher promotion of wound healing as compared to the acceleration effect of chitosan. Moreover, comparison between the routes of administration of MSCs was done and revealed that intradermal injection of MSCs was more effective than systemic injection in wound healing. Intradermal injection of MSCs was proved to be an efficient and reliable mode of treatment of wound areas. They induced rapid cellular proliferation, granulation tissue formation, neoangiogenesis, collagen fibres synthesis and early complete wound healing.

## Supporting Information

S1 Chart Mean values of wound area in excisional wounds of different groups. (JPG)

S2 Chart Mean values of epidermal thickness in different groups. (JPG)

S3 Chart Mean values of area percent of collagen fibres in different groups. (JPG)

S4 Chart Mean values of area percent of CD31 in different groups. (JPG)

S1 Table Mean values of wound area in (cm^2^) ± standard deviation and percent of reduction (in parentheses) in excisional wounds of different groups. (JPG)

S2 Table Mean values, standard deviation and significance of epidermal thickness in different groups. (JPG)

S3 Table Mean values, standard deviation and significance of area percent of collagen fibres in different groups. (JPG)

S4 Table Mean values, standard deviation and significance of area percent of CD31 in different groups. (JPG)
