# Supplementary figures and images for: The Effect of Mesenchymal Stem Cells and Chitosan Gel on Full Thickness Skin Wound Healing in Albino Rats: Histological, Immunohistochemical and Fluorescent Study
Source: PLoS One. 2015 Sep 24;10(9):e0137544. doi: 10.1371/journal.pone.0137544 (PMC4581728; doi:10.1371/journal.pone.0137544)

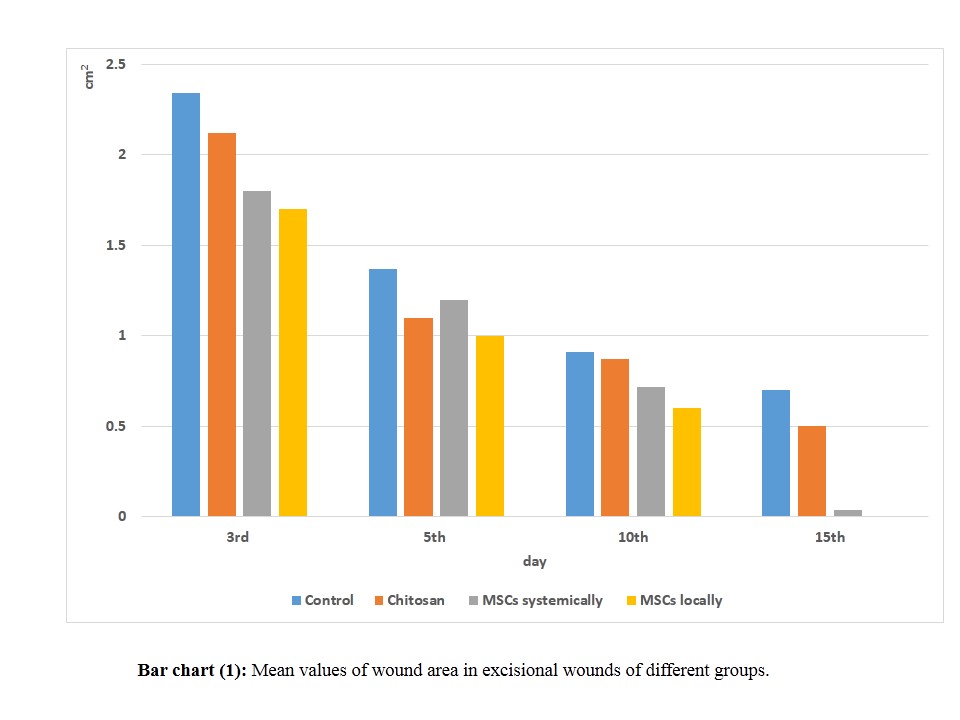

Supplement: S1 Chart — (JPG) [file pone.0137544.s001.jpg]

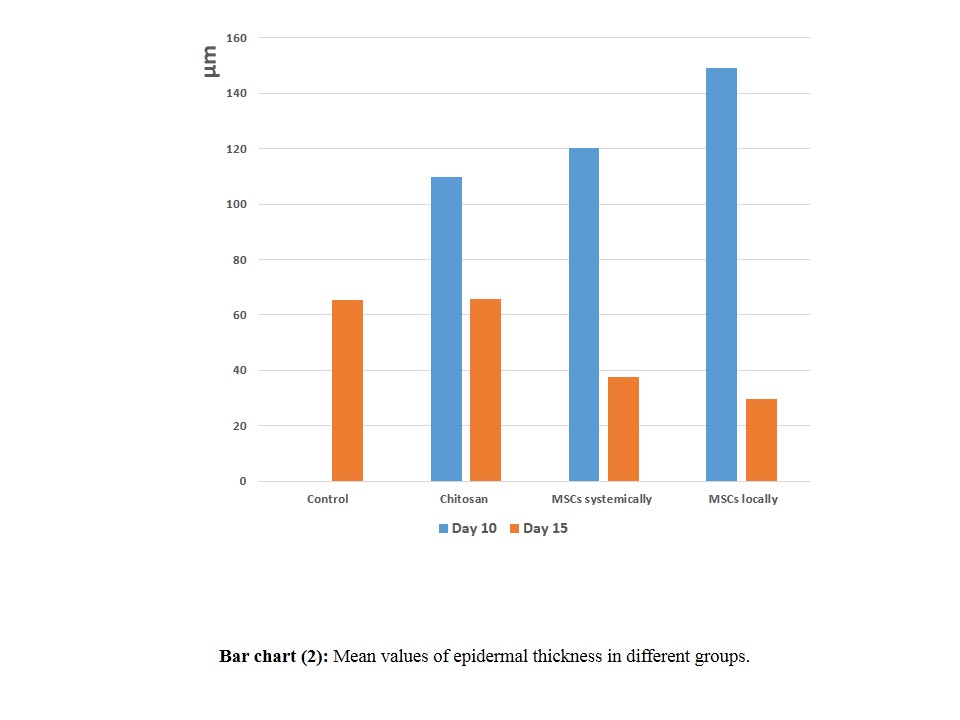

Supplement: S2 Chart — (JPG) [file pone.0137544.s002.jpg]

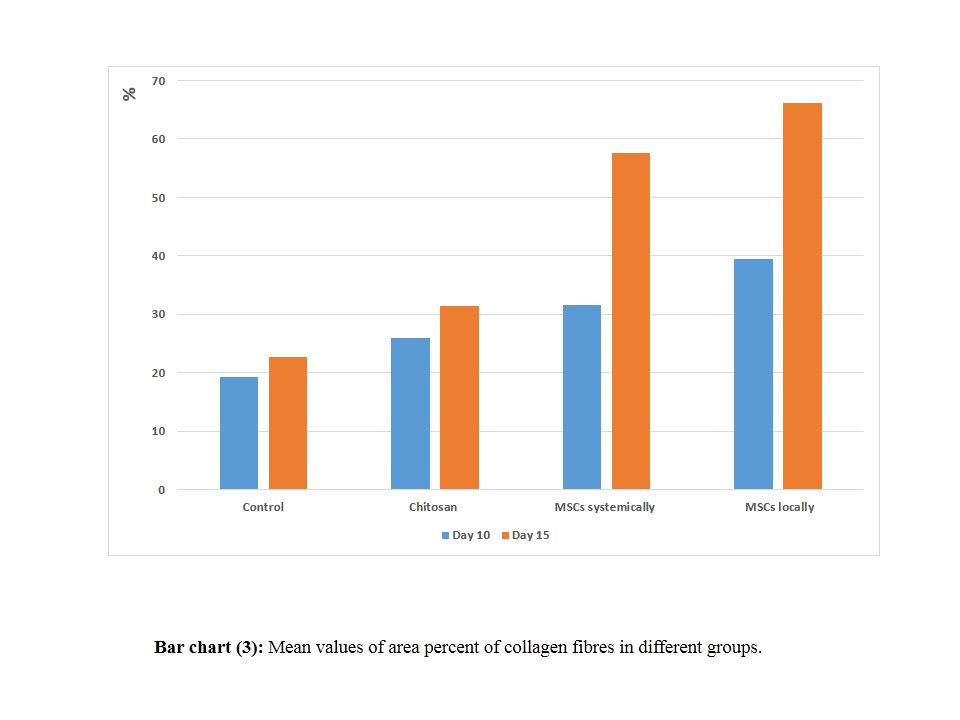

Supplement: S3 Chart — (JPG) [file pone.0137544.s003.jpg]

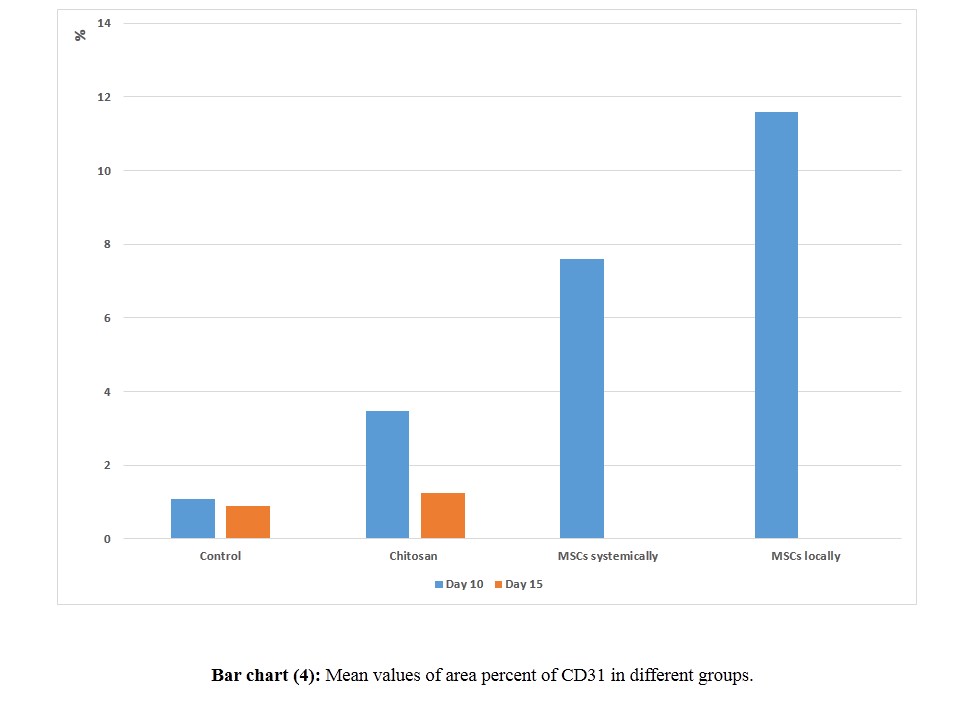

Supplement: S4 Chart — (JPG) [file pone.0137544.s004.jpg]

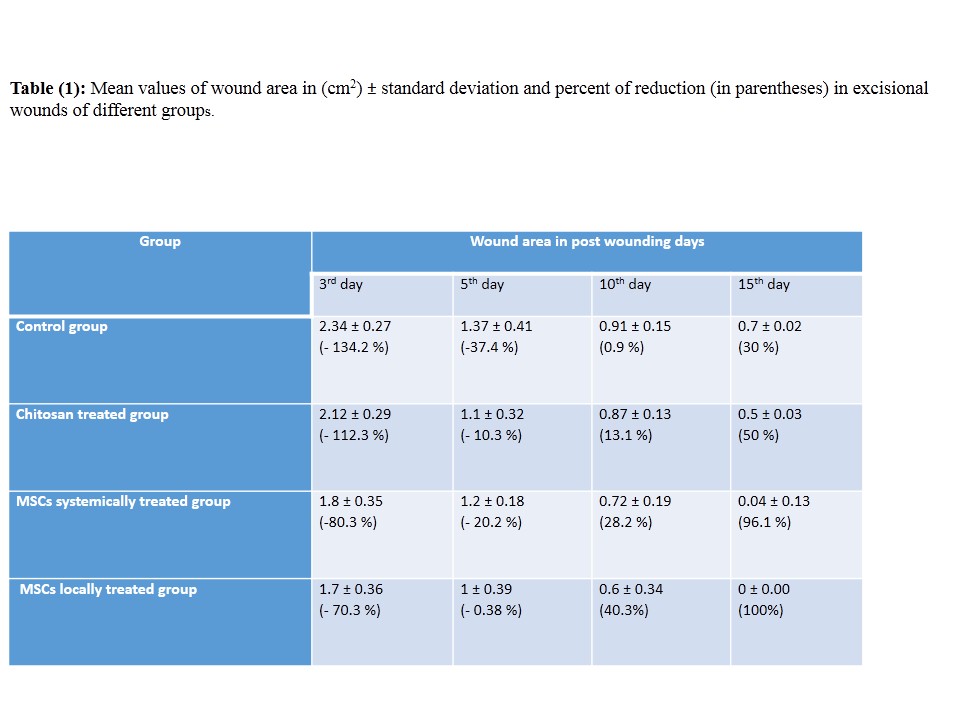

Supplement: S1 Table — (JPG) [file pone.0137544.s005.jpg]

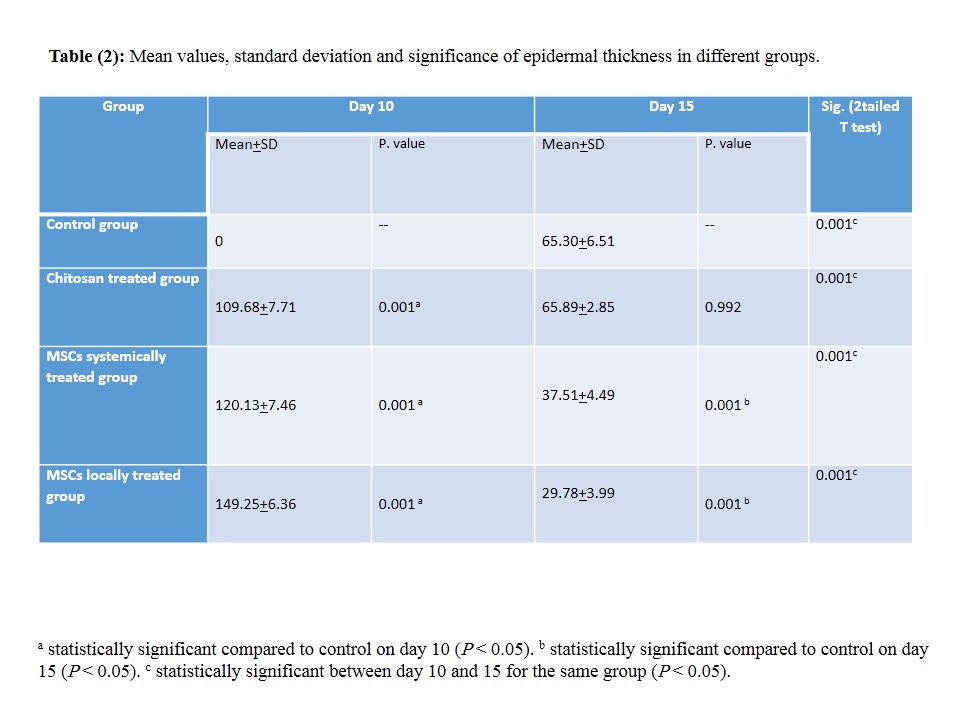

Supplement: S2 Table — (JPG) [file pone.0137544.s006.jpg]

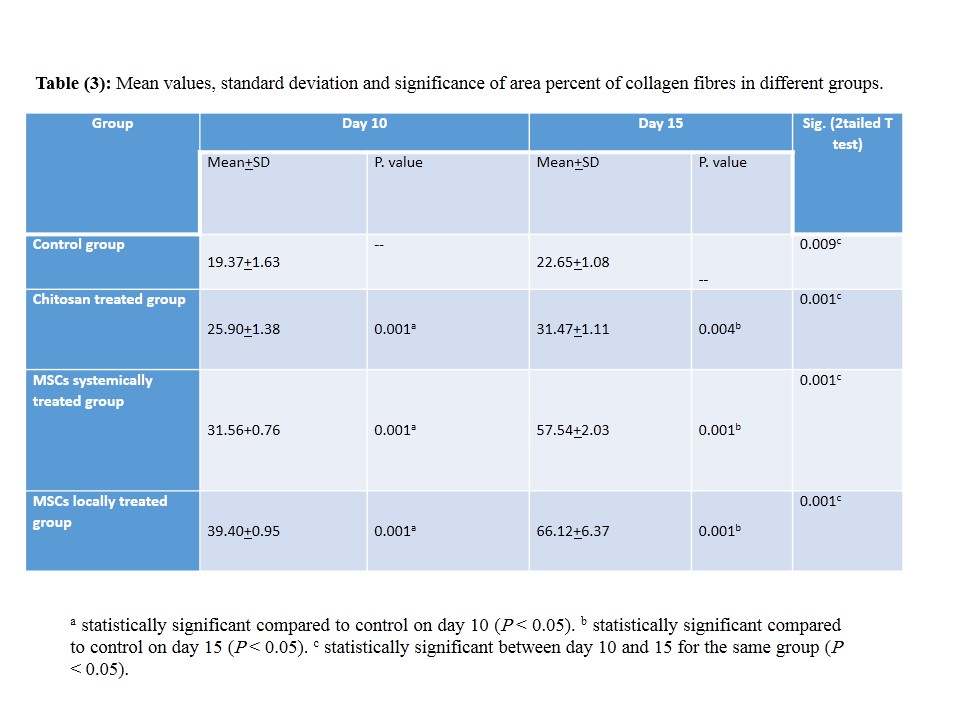

Supplement: S3 Table — (JPG) [file pone.0137544.s007.jpg]

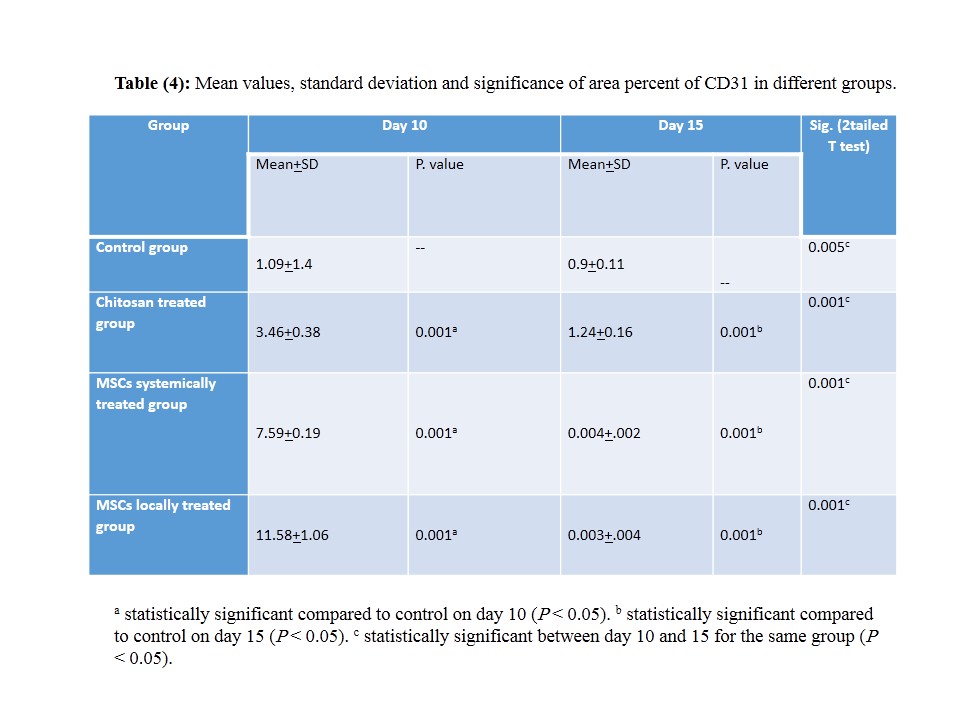

Supplement: S4 Table — (JPG) [file pone.0137544.s008.jpg]
